# Performance Assessment of a Piezoelectric Vibration Energy Harvester for Hybrid Excitation with Varying Cross Sections

**DOI:** 10.3390/s24237629

**Published:** 2024-11-28

**Authors:** Bartłomiej Ambrożkiewicz, Zbigniew Czyż, Vikram Pakrashi, Jakub Anczarski, Paweł Stączek, Andrzej Koszewnik, Mirosław Wendeker, Grzegorz Litak

**Affiliations:** 1 Faculty of Mathematics and Information Technology, Lublin University of Technology, Nadbystrzycka 38, 20-618 Lublin, Poland; b.ambrozkiewicz@pollub.pl; 2 Faculty of Aviation, Polish Air Force University, Dywizjonu 303 Street No 35, 08-521 Dęblin, Poland; z.czyz@law.mil.pl; 3 UCD Centre for Mechanics, School of Mechanical and Materials Engineering, D04 V1W8 Dublin, Ireland; 4 Faculty of Mechanical Engineering, Lublin University of Technology, 36, 20-618 Lublin, Poland; jakub.anczarski@pollub.edu.pl (J.A.); p.staczek@pollub.pl (P.S.); m.wendeker@pollub.pl (M.W.); g.litak@pollub.pl (G.L.); 5 Faculty of Mechanical Engineering, Białystok University of Technology, Wiejska 45C, 15-351 Białystok, Poland; a.koszewnik@pb.edu.pl

**Keywords:** hybrid energy harvesting, piezoelectric sensor, wind tunnel, mechanical vibrations, airflow, computational fluid dynamics

## Abstract

This paper experimentally examines the influence of hybrid excitation on the performance of vibrational piezoelectric energy harvesting systems on a bluff body with a variable cross section along its generatrix. A combination of vibrational excitation from a shaker and airflow is considered the source from which energy is harvested. Varied excitation frequencies and airflow velocities across five different masses were considered, each defining the natural frequency of the system. The system’s performance in hybrid excitation, enhancements in energy harvesting, and challenges with these was observed, helping to determine optimal operating conditions to function effectively in ambient environments. The tests identified the conditions and ranges within which maximized harvesting responses were observed. Next, computational fluid dynamic (CFD) simulations were carried out to understand the impact of circular and square cross sections controlling the nature of the airflow and representative of the wide range of cross sections that may be utilized for such purposes. The analyses helped contextualize the opportunities and limitations of the use of such cross sections and helped in understanding if a transition from one cross section to another can lead to an assimilation of the advantages observed in using each cross section independently.

## 1. Introduction

Energy harvesting (EH) is an expansive field focusing on converting energy from the external environment that is gaining traction in practical applications [1,2,3]. Miniaturized systems serve as effective independent power sources for autonomic sensors and other low-power electronic devices in inaccessible areas like open water [4], planetary exploration [5], or mines [6]. EH aligns with a modern green technology philosophy, utilizing natural energy sources such as solar [7], wind [8], or sea waves [9] in an environmentally friendly manner, even though piezoelectric systems often contain heavy metals.

One of the primary challenges in developing EH systems is maximizing their efficiency and adaptability to varying external excitations [10,11]. To achieve maximum output power, these systems are typically designed to operate at their resonance frequency. However, in real-world environments, there is no single frequency acting on the system [12]. Instead, different frequency components are observed in the spectra as additional peaks or super- or sub-harmonics. The use of nonlinear systems [13], collocated harvesters, or multiple sources of excitation can be particularly relevant, but most harvesting systems are often linear. Also, their applicability often comes from a monitoring perspective, where the linearity of the device makes such objectives easier [14]. Another challenge lies in identifying and controlling the optimal operating conditions from multiple excitations to ensure the longest possible lifetime of the EH system [15,16]. The analysis of hybrid excitation has garnered significant interest from researchers [17,18]. Hybrid excitations in piezoelectric energy harvesting systems utilize both mechanical vibrations and airflow-induced excitations to enhance energy conversion. This combined approach allows the system to harness a broader range of environmental energy sources, leading to higher overall power output and efficiency. By integrating these dual excitation mechanisms, the system can achieve better performance and reliability in variable conditions. While there have been hybrid systems proposed in the literature, the characterization and advantages of such systems are strongly a function of the needs and the range of ambient excitation within which such performance is assessed. In this regard, there is a range of setups, characteristics, applications, and insights that can aid the applications of energy harvesting.

In line with this trend, an innovative concept of a hybrid energy harvesting system based on the piezoelectric effect is proposed [19] in this paper. To analyze system performance, wind-induced broadband vibrations and mechanically induced harmonic vibrations were applied to identify the optimal conditions for energy harvesting and the most effective design, taking into account various masses. The experiments consider a transition of the cross section over the length. Wind excitation is characterized by its possibility to create rich dynamics and related instabilities, including vortex-induced vibration (VIV), flutter in airfoils, galloping in prismatic structures [20], and systems composed of multiple resonators, where one bluff body affects the excitation of another [21]. Additionally, research by Wang et al. [22] and Yang et al. [23] explored the effects of mixed cross sections of bluff bodies, finding that a circular cross section favors VIV while a square cross section causes galloping phenomena. Building on this work, this paper proposes a modified geometry for a bluff body, featuring a cross section that transitions along its length from a circle to a square. Subsequently, the paper also investigates if a transition can assimilate the advantages that may be available from using each cross section individually, by carrying out extensive computational fluid dynamic (CFD) simulations. The mixed design of the bluff body with varying cross-sectional shapes and different masses is experimentally investigated. The circular body is sensitive to vortex-induced vibrations [24], while the square body is prone to the galloping effect [25]. In structures like chimneys, towers, high-voltage lines, or bridge decks, countermeasures are often taken to avoid these self-excited structural vibrations. However, in aviation [26,27] and energy harvesting applications [28,29], these effects can positively impact the dynamic response. Increased vibrations during self-excited oscillations tend to excite the system with high amplitudes, directly enhancing power output.

Several studies have compared the performance of energy harvesting from a combination of excitation sources, sometimes mixing excitation sources, and for a range of technologies. Zhou et al. investigated harvesting from low-speed wind energy with a bi-stable piezoelectric energy harvester from vortex-induced vibration and galloping [30] with validations on a square-sectioned bluff body, emphasizing the need for simple and low-cost harvesters for such applications. A piezoelectric harvester with magnetic field coupling indicates a similar need [31]. Harvesting from wind excitation has been considered experimentally with a bluff-body low-speed wind turbine [32,33] and also in subsequent analysis on extreme value excitation and its use in wind effect monitoring [34]. A dual-source vibration energy harvester with a spherical pendulum to capture both flow-induced and multi-directional vibration energies integrating a specialized bluff body with a square section attached to the end of a piezoelectric beam has been validated, demonstrating the nonlinear behavior of both [35]. While the investigation of wind-induced vibration energy harvesting [36,37] and its accentuation by hybrid sources of harvesting [38] or accentuation utilizing internal resonances [39] have been popular [40,41], there are fewer experimental studies on different wind speeds combined with hybrid excitation and for different cross sections of airflow. The variability of the shape of the bluff body leads to a lock-in phenomenon coming from coupled vortex-induced vibration and galloping, which significantly improves the voltage output while ensuring robust performance across a range of aerodynamic parameters and uncertainties [42]. In the research of Yan et al. [43], hybrid excitation increased the ability of the harvester to enhance its energetic potential by leveraging the interaction between base and galloping excitations, allowing for the emergence of different nonlinear phenomena that can be optimized for maximum power output with minimal transverse displacement.

These advantages of hybrid excitation and different bluff-body shapes motivated us to design a piezoelectric energy harvester with varied shapes along its generatrix (square to circle diameter) mounted on a cantilever beam. The first design has been presented in detail in [44], in which the system operated in the airflow at higher airflow velocities. Here, the harvester is redesigned and equipped with additional springs to obtain the oscillating solutions from lower velocities of airflow. In the current work, the tests are extended to a wide range of frequencies of excitation and airflow velocities using two sources of excitation. In this case, quantitative analysis is conducted to compare the impact of the single and double sources of excitation. The requirement of simple harvesters for wind flow harvesting along with other narrowband excitations is less studied, especially experimentally, but it is observed that there is a need for such performance assessment for these types of devices and excitation scenarios. This paper addresses the need for a piezoelectric harvester for a range of wind flow excitations and sinusoidal vibrations from a shaker, allowing for a hybrid excitation for such performance comparison. To optimize performance, this study coupled hybrid excitation with variable airflow velocity in a wind tunnel and external mechanical vibrations from a shaker. In the current design, the system provides the oscillating solution in a wide range of frequencies of excitation, even outside its characteristic frequency, which was one of the motivations for its redesign. Additionally, an initial analysis involving flow term calculation was conducted, and the output voltage from the piezo element, along with the acceleration of the tip mass, was measured for different flow velocities and excitations. Additionally, a CFD analysis was conducted on how the lifting force changes and acts on bluff bodies of different shapes. To emphasize and sum up this research, we can state that it uniquely explores a hybrid excitation approach, combining broadband wind excitation with narrowband harmonic excitations to enhance piezoelectric energy harvesting. The study introduces a new design with transitioning bluff-body geometries, enabling the assessment of circular and square cross sections under both experimental and CFD conditions. The findings demonstrate that dual excitation significantly boosts output voltage and expands the operational frequency range, offering critical insights into the effectiveness of shape and excitation combinations for optimized energy harvesting.

The remainder of this paper is structured as follows. Section 2 describes the design of the energy harvester and test rig. Section 3 outlines the experimental procedure and specifies the experimental cases, additionally discussing the data processing and presenting voltage results in 3D plots. In Section 4, the CFD model is presented, while in Section 5, the results obtained from the CFD calculations are described. The Section 6 summarizes the research findings.

## 2. Energy Harvesting System and Experimental Setup

**Figure 1:**
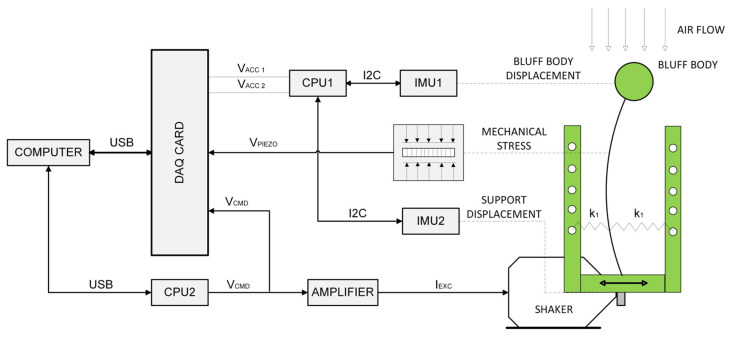
Diagram of the measurement system.

Figure 1 schematically illustrates the measurement system and the elements of the test object. The system utilizes two excitation sources: mechanical vibrations from a shaker and airflow within a closed-section wind tunnel. The energy harvester converts these vibrations into electrical energy via a piezoelectric sensor mounted on a cantilever beam, where mechanical strain influencing the sensor generates electrical output. This process enables efficient energy harvesting from combined vibrational and aerodynamic forces.

In terms of the communication and data acquisition part of the experimental setup, the desired sine wave excitation with defined frequency on the shaker was generated by a 32-bit microcontroller (CPU2) by a control sequence composed in Matlab Simulink. Next, the digital amplifier supplied the shaker with the excitation current I_EXC_ proportional to the voltage V_CMD_. Accelerations of the shaker and bluff body were measured using MEMS accelerometers with range of ±8 g and a 12-bit resolution at 800 Hz (IMU 1 and IMU 2). The acceleration signals were transferred to another microcontroller (CPU1) via the I^2^C communication protocol and converted to analogue values (V_ACC_ 1, V_ACC_ 2), then they were transferred to the data acquisition card. All abovementioned signals were digitally recorded at 800 Hz using the data acquisition software FlexLogger (2022 Q2 version).

**Figure 2:**
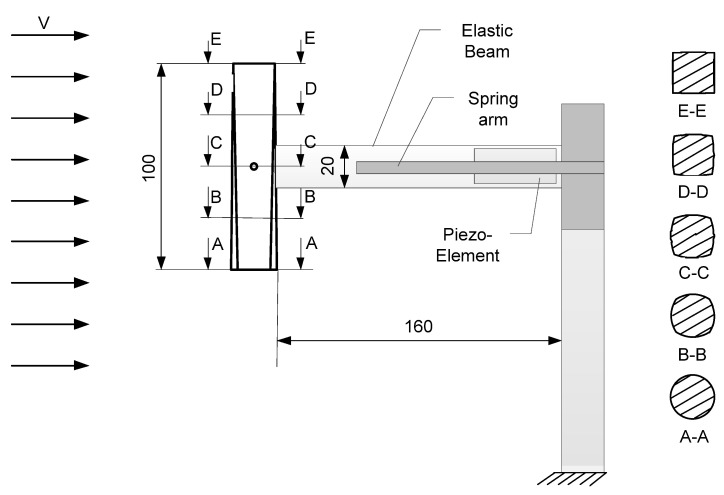
Bluff-body geometry with variable cross section along its generatrix.

The piezoelectric system comprises an aluminum elastic beam, a piezoelectric element, a bluff body, and a spring arm. The arm is used to attach springs, with their other ends connected to the elastic beam. The elastic beam, with a total length of 200 mm, has a cross section measuring 20 mm wide and 1 mm thick (Figure 2). The free span of the beam, not including the attachment points, measures 160 mm (plus 20 mm for the attachment to the stinger and 20 mm for the bluff body). The beam is bolted to a support structure, which is a vertical aluminum flat bar with a cross section of 20 mm width and 4 mm thickness.

The bluff body spring arm is printed from PLA (polylactide) material using 3D printing technology and is bolted to the support structure at the height where the bluff body acts. The orientation of the bluff body and the elastic beam allows for horizontal oscillation, a positioning necessary due to the vortices induced behind the bluff body.

Vortices, and thus vibrations, are induced by a bluff body composed of a cylinder and a cuboid. The geometry was designed using Solidworks CAD software (SP3.1) and next printed with a 3D printer using the fused deposition modeling (FDM) method. For the needs of the experiments, the 5 different bluff bodies were printed with various infill, defined in the 3D printing slicer software UltiMaker Cura 4.3. The masses were ordered according to infill with steps of 20% (from 20% to 100%), which automatically defined the masses (detailed information will be presented in next section. The bluff body’s cross-sectional shape transitions smoothly along its entire length from a square to a circle using the spline function. Notably, the cross sectional areas of both the square and circle at the ends are identical, each measuring 400 mm^2^. Figure 2 illustrates the selected cross sections of the bluff body and its position within the measurement space of the wind tunnel. The bluff body is 100 mm high.

**Figure 3:**
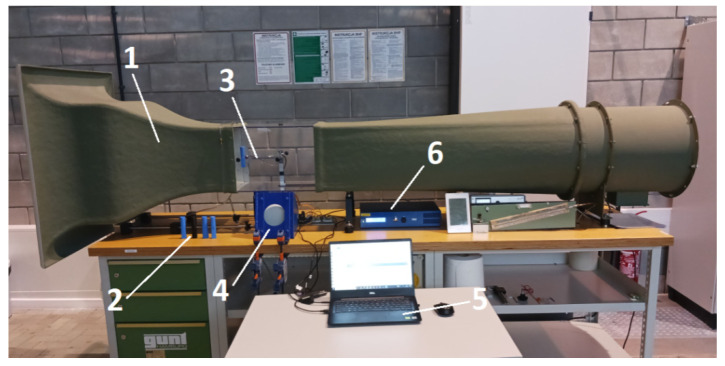
Overview of the experimental test rig: (1) HM 170 open wind tunnel (GUNT Hamburg), (2) set of studied bluff bodies, (3) piezoelectric energy harvesting system, (4) vibration generator, (5) computer with data acquisition software, (6) digital amplifier.

A GUNT HM 170 open wind tunnel with a closed measurement area was used in the experiment, described in detail in [44]. The wind tunnel’s measurement section has a square cross section of 300 mm × 300 mm, with the test object positioned at the center. The airflow velocity around the object was controlled by adjusting the speed of a fan installed at the wind tunnel outlet. Figure 3 shows the test object and the whole test rig, starting with the wind tunnel and finishing on the piezoelectric energy harvester.

To excite the elastic beam, a TIRA S513 vibration generator was placed beneath the measurement section. This device has a rated peak force of 100 N and a frequency range of 2 to 7000 Hz, enabling an axial displacement of 13 mm, a maximum velocity of 1.5 m/s, and a maximum acceleration of 45 g. The sinusoidal excitation signal was amplified during the measurements using a TIRA DA 200 digital amplifier (TIRA, Schalkau, Germany).

**Figure 4:**
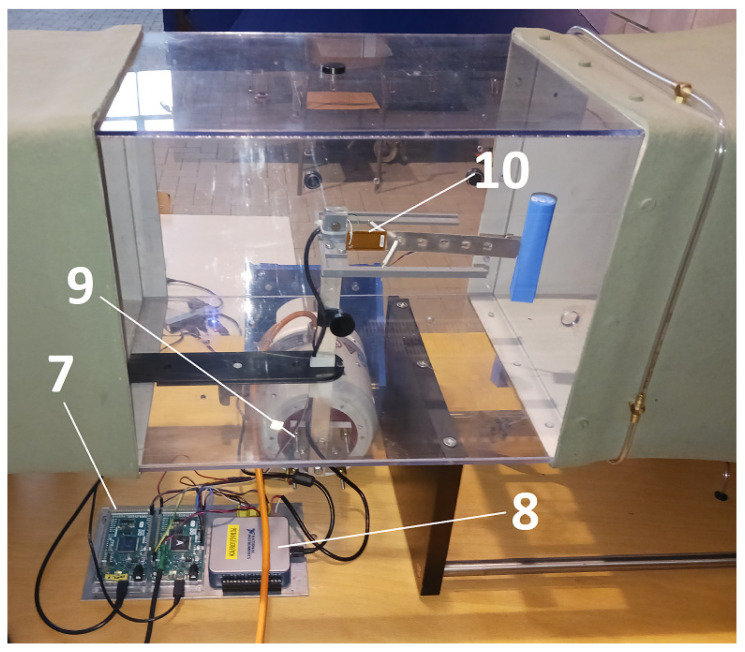
Overview of the experimental test rig: (7) microcontrollers (Arduino DUE boards), (8) data acquisition card, (9) accelerometer mounted on the shaker, (10) piezoelectric sensor.

This research continues from the study discussed in [44], which also focused on the same bluff body, but mounted on a freely moving elastic beam. In this experiment, the movement of the elastic beam was modified by attaching springs to both sides (Figure 1, Figure 2, Figure 3 and Figure 4).

## 3. Analysis of Experimental Results

Voltage generated by the proposed energy harvesting system in the previous section subjected to hybrid excitation was investigated. Hybrid excitation was induced by varying airflow velocities and external sinusoidal inputs. Five different bluff bodies with varying masses were analyzed. The sinusoidal excitation was generated using a vibration generator connected to the support structure, while the airflow velocity was adjusted by modifying the fan’s rotational speed in the test section. During the experiment, the voltage generated by the piezo element and the acceleration of the shaker were recorded. The tests were divided into two stages, i.e., at first, the tests were conducted only with the application of the shaker, and next the tests were conducted by hybrid excitation in the form of the shaker and airflow tunnel.

**Figure 5:**
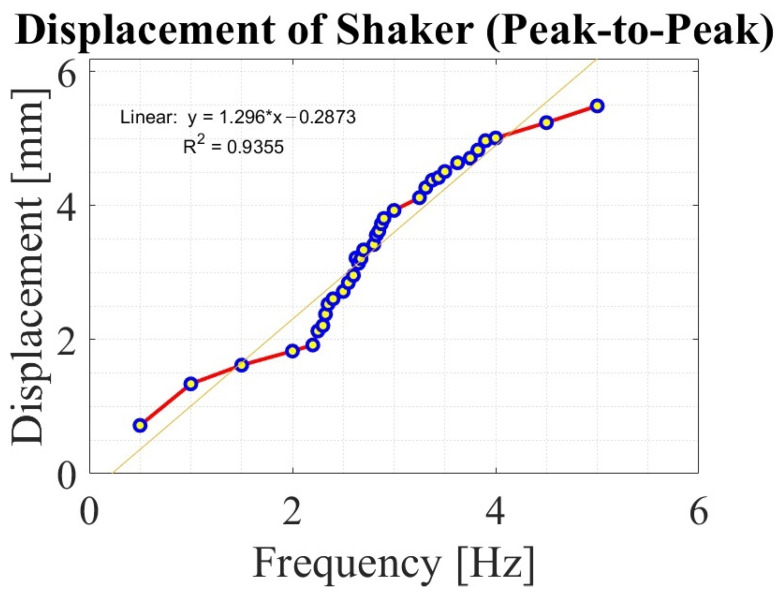
Frequency of excitation–displacement characteristics of the shaker.

In Figure 5, the results of displacement *x* of the shaker are collected by the specific frequency of excitation *f*. The results obtained are from the processing of the acceleration signal collected from the accelerometer mounted on the exciting tip of the shaker. The range of operating frequencies for all the masses is 0.5 Hz to 5 Hz, with an increased number of registered points around the characteristic frequencies for each mass of the bluff body. Considering integration error, the characteristics of the shaker have an almost linear trend and Pearson’s linear correlation coefficient is close to unity, showing the strong correlation between the frequency of excitation and the displacement of the shaker. Table 1 shows detailed displacement data for various frequencies of excitation.

**Figure 6:**
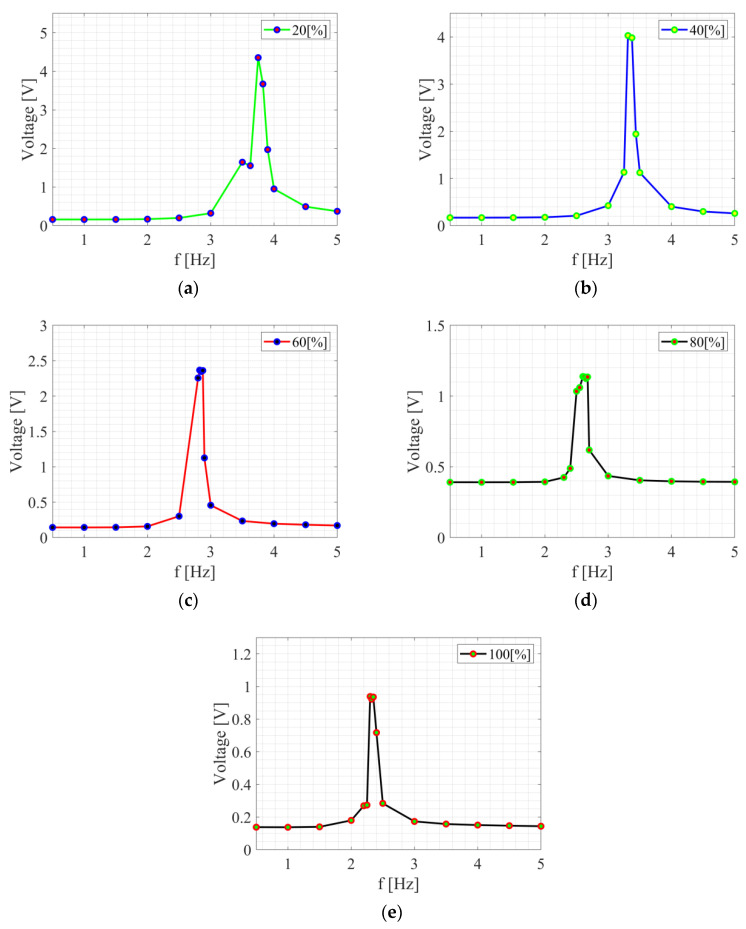
Resonance curves obtained for bluff bodies of different infill percentage: (**a**) 20%, (**b**) 40%, (**c**) 60%, (**d**) 80%, and (**e**) 100%. The larger mass of the bluff body results from the lower natural frequency.

First, a linear analysis was carried out to define the range of operating frequencies for each bluff body. In Table 2, the list of bluff-bodies applied in the experiments are listed by defining their mass and the range of resonance frequencies obtained experimentally. The resonance curves were derived (Figure 6) by calculating the root mean square (RMS) values of the generated voltage signals. Measurements were carried out without airflow in the wind tunnel. By analyzing data on the RMS dependence of voltage on frequency for m_1_ = 20%, it can be observed that for the low-frequency range (0.5–2.5 Hz) the values of the voltage are relatively constant and low, oscillating from 0.15 to 0.19 V. This suggests that in this frequency range, excitation does not have a significant effect on generated voltage. In the mid-frequency range (2.5–3825 Hz), the results begin to increase with frequency. A particularly large increase can be observed at 3.5 Hz (1.64 V) and 3.625 Hz (1.55 V), up to a peak value at 3.75 Hz (4.35 V). After this point, the voltage decreases, but is still higher than at lower frequencies. After reaching its peak at 3.75 Hz, the RMS value of voltage decreases, reaching 0.95 V at 4 Hz and further decreasing to 0.37 V at 5 Hz. This may suggest suppression of excitation effects at higher frequencies. It can therefore be concluded that the frequency of the exciter has a significant effect on output voltage, especially from 2.5 Hz to 4 Hz, where we observe a significant increase in the generated voltage.

Analogously, for bluff-body m_2_ = 40%, the RMS voltage in the frequency range of 0.5 Hz to 2.5 Hz is relatively constant and low, oscillating from 0.16 V to 0.20 V. The values are stable, suggesting that low excitation frequencies have little impact on the RMS voltage. In the medium-frequency range, i.e., from 3 to 3.5 Hz, significant changes in RMS voltage are observed, where at 3 Hz it is 0.42 V, and the maximum is reached between 3.3125 Hz (1.22 V) and 3.375 Hz (1.21 V), indicating strong excitation in this frequency range. After these peak values, the voltage begins to decrease, suggesting damping of excitation effects.

For m_3_ = 60%, in the low-frequency range (0.5–2.5 Hz), the RMS voltage is relatively stable, oscillating around 0.15 V. A significant increase is observed at 2.5 Hz, where the RMS value is 0.30 V. In the medium frequency range (2.5–3 Hz), there is an increase in RMS voltage, with the system reaching near-peak values: 2.26 V at 2.8 Hz, 2.36 V at 2.825 Hz, 2.35 V at 2.85 Hz, and 2.36 V at 2.875 Hz. The voltage begins to decrease above 2.875 Hz, reaching 1.13 V at 2.9 Hz and 0.45 V at 3 Hz. The RMS voltage continues to decrease from 3 Hz to 5 Hz, reaching values from 0.23 V to 0.17 V. The decrease is steeper after reaching the peak in the medium-frequency range.

For m_4_ = 80%, in the first frequency range (0.5–2.3 Hz), the RMS voltage is relatively stable and low, oscillating around 0.39 V to 0.42 V. In the medium-frequency range (2.3–3 Hz), the RMS voltage starts to increase from around 2.4 Hz, reaching a value of 0.48 V, and at 2.5–2.675 Hz, it reaches peak values up to 1.14 V at 2.6 Hz. After reaching the maximum value, the voltage drops sharply to 0.61 V at 2.7 Hz and further to 0.44 V at 3 Hz. Above 3 Hz, the RMS voltage is relatively stable and low, around 0.4 V.

For the maximum mass m_5_ = 100%, the increase in RMS voltage begins at about 2 Hz, reaching a maximum in the range of 2.3–2.35 Hz, and then decreases. After reaching the maximum value, the voltage drops to a level similar to the values in the low-frequency range. At low and high frequencies (below 2 Hz and above 3 Hz), the RMS voltage remains at a relatively constant level.

From the above analysis, it follows that the highest resonance frequency (3.75 Hz) is achieved by mass m_1_, for which the maximum of generated voltage is 6.68 V. The lowest resonance frequency (2.3 Hz) is with mass m_5_, for which the maximum voltage is 2.04 V.

**Figure 7:**
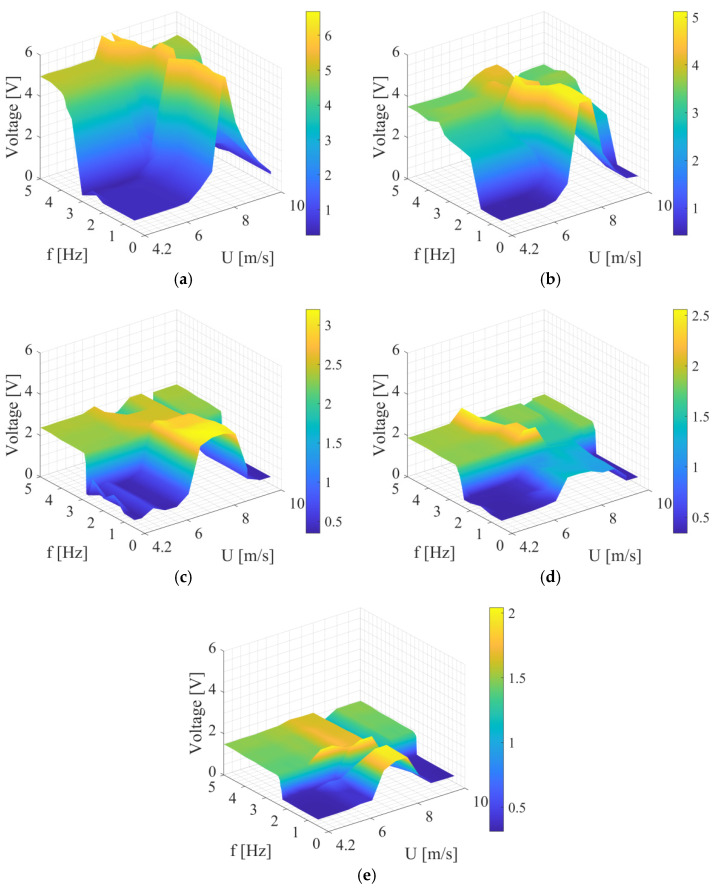
RMS voltage results by the hybrid excitation for studied bluff bodies, infill percentage: (**a**) 20%, (**b**) 40%, (**c**) 60%, (**d**) 80%, and (**e**) 100%. The non-monotonic shape of the voltage output with a single maximum in the limit of f = 0 Hz indicates that the leading effect is VIV with frequency lock around the natural frequency of the bluff-body spring system. The higher level of the voltage output above the natural frequency of shaker excitation is closely related to the faster left–right motion of the bluff body. Beyond this limit, the vortex shedding frequency is defined by shaker vibration excitation.

Next, the experiments considered an additional source of excitation in the form of airflow along with mechanical excitation from the shaker in the range of previous frequencies. The range of airflow velocity was from 4.2 m/s to 10 m/s based on previous research [44] and the need for the system’s operating under the least possible energy supply for the excitation. The visualization of the results obtained is presented in Figure 7, in which the results of RMS voltage values are depicted. In the surface plots, it can be observed that the energy harvester operates with oscillating solution in the wider range of operating frequencies and airflow velocities. Additionally, by the hybrid excitation, the highest values of generated voltage are obtained by lower frequency of excitation due to mixing of the sources of excitation. At first glance, it can be observed that hybrid excitation improves the performance of the system.

In Table 3, the results of the highest obtained voltage *V* are collected by the specific frequency of excitation *f* and specific airflow velocity *U*. Based on the results, it can be stated the optimal airflow velocity range is 7–8 m/s. Referring to Figure 7, with the airflow velocities mentioned, the oscillating solution is observed. In Table 4, the results of harvester’s performance are collected.

The analysis of the results presented in Figure 7 indicates system-tested lower performance for low wind speeds and low excitation frequencies with the shaker, which is expected. However, this contradicts the results from the literature, which illustrate the effective operation of systems for lower velocities, such as in offshore turbines [45,46]. Since vortex-induced vibrations (VIVs) should play a significant role in energy harvesting, control and efficacy of excitation sources controlling the flow of wind is an important aspect to investigate when working in a hybrid manner with mechanical excitation. Here, two widely varying cross sections that may be used for such excitation are a circle and a square. Apart from their respective efficacy, there is also the question of whether their combination into a single system (Figure 2), as considered in the experiments, brings an advantage over individual advantages and disadvantages of the respective cross sections. This was addressed through a computational fluid dynamic (CFD) implementation, where shapes were modeled separately, keeping their characteristic dimensions.

## 4. CFD Numerical Model

Computational fluid dynamics (CFD) [47,48] was used to analyze airflow effects on the piezoelectric setup, allowing for enhancing device geometry and energy capture. CFD also helps in visualizing pressure distribution and turbulence, which are crucial for maximizing vibration-induced energy generation [49,50]. Additionally, it reduces the need for costly experimental prototypes, saving time and resources during the design process.

**Figure 8:**
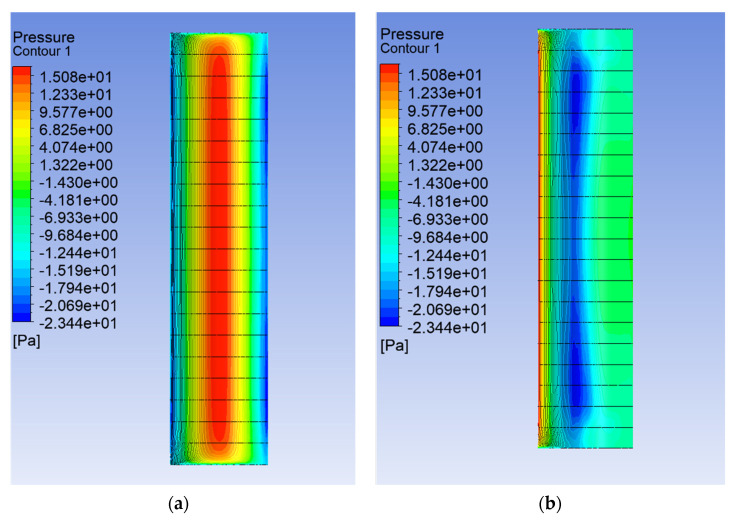
Pressure distribution on the surface of the cylinder under testing: front (**a**) and side (**b**) views.

Two different geometries were considered in this work: a circular and a square cross section. The model was created by reflecting the dimensions of the measurement space of the wind tunnel used for the experiment. The domain width in both cases was 300 mm and the length 450 mm. The height of the measurement space in the tunnel was also 300 mm, but to shorten the calculation time, the model assumed analysis until a height of 5 mm, the boundary condition of symmetry was applied on both sides of the domain, and it was possible to rapidly determine the forces acting on the walls of the tested shape. Assuming that the test object is 100 mm high, the obtained force values should be multiplied 20 times. This assumption was made based on calculations conducted for an example velocity of 5 m/s. The cylinder model, with a height of 100 mm, was divided into 5 mm sections, as shown in Figure 8, and calculations were performed. Force values for each section were obtained. This distribution allowed for the determination of the total force acting on the lateral surface of the cylinder. It was demonstrated that the force acting on a selected single section from the central part multiplied by 20 differed by 9.85% from the actual total force obtained by summing the forces from all sections. For the calculations carried out, this value was considered a sufficient approximation, also taking into account the time savings.

**Figure 9:**
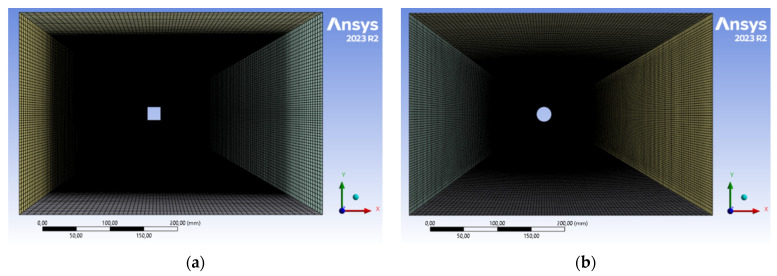
General view of calculation grids for individual models: (**a**) square, (**b**) circle.

**Figure 10:**
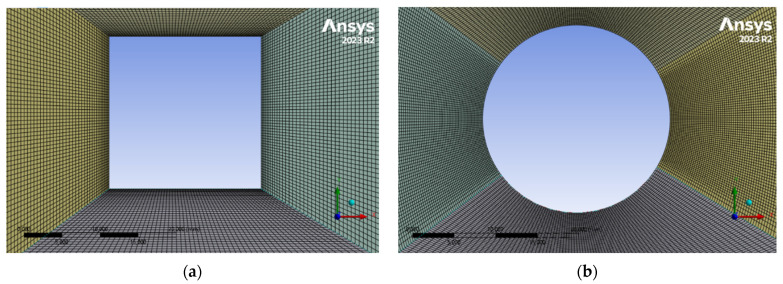
Detailed view of computational grids in the area of the examined object: (**a**) square, (**b**) circle.

Next, meshes using hexahedral elements were generated for the cross sections under consideration via the Ansys Meshing module, selecting the appropriate element sizes for the characteristic dimensions of the computational domain. The maximum value of the skewness coefficient for the generated meshes was 0.67 and 0.76 for the square and circle, respectively. The total number of elements for the circular cross section was 338,400 and for the square cross section, it was 552,600 elements. When creating the mesh, the same element size was assumed on the surfaces of the tested object. A pictorial view of the meshes in computing machines is shown in Figure 9 and Figure 10.

The calculations were performed for three-dimensional, turbulent flow in an unsteady state—transient with Ansys Fluent software (2023R2), which is based on the finite volume method. The semi-implicit method for pressure-linked equation (SIMPLE) was selected for pressure-related equations to determine the relationship between pressure and velocity. First, calculations for minimum and maximum velocities were carried out for two viscous models, i.e., laminar and LES. As the results converged, it was decided to carry out further calculations for the laminar model. This model was chosen because the calculations represent a preliminary analysis and the laminar model is less computationally expensive. Moreover, the Reynolds number used ranges from 0.7 × 10^3^ to 1.4 × 10^4^, which for flow around bodies, allows for this type of modeling. Subsequently, boundary conditions were assigned. The CFD geometric model has one inlet and one outlet. The inlet was set as a velocity inlet with values ranging from 0.5 m/s to 10 m/s (Table 5). The outlet from the domain was set as a pressure outlet, and the outlet pressure was standard atmospheric pressure. Physical parameters such as air density and viscosity were set at a temperature of 288 K and were 1.2257 kg/m^3^ and 1.7965 × 10^−5^ Pa·s, respectively.

## 5. Results of CFD Numerical Model

**Figure 11:**
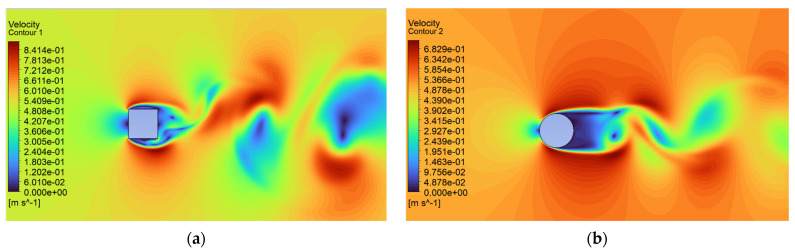
Velocity contour around the examined fixed bluff-body object for U = 0.5 m/s: (**a**) square, (**b**) circle.

**Figure 12:**
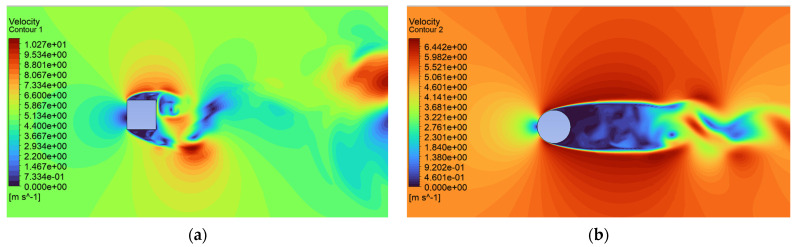
Velocity contour around the examined fixed bluff-body object for U = 5.0 m/s: (**a**) square, (**b**) circle.

**Figure 13:**
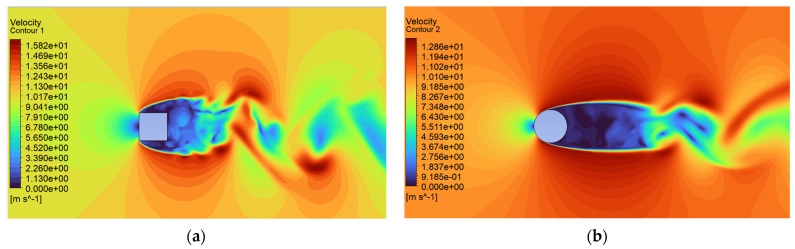
Velocity contour around the examined fixed bluff-body object for U = 10.0 m/s: (**a**) square, (**b**) circle.

**Figure 14:**
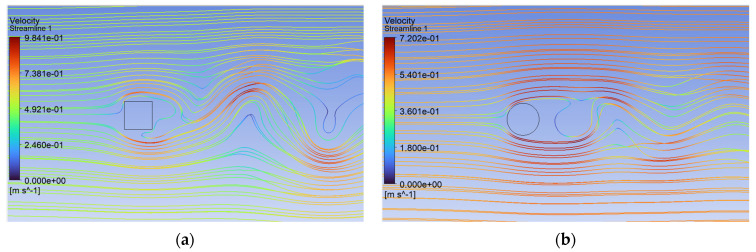
Streamlines around the examined fixed bluff-body object for U = 0.5 m/s: (**a**) square, (**b**) circle.

**Figure 15:**
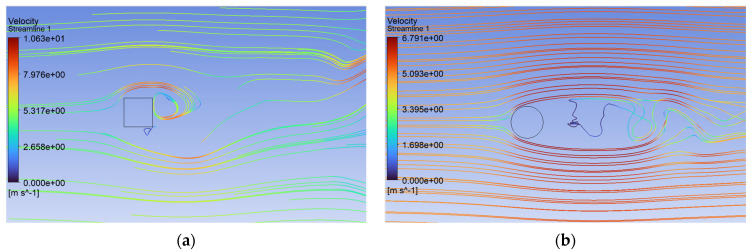
Streamlines around the examined fixed bluff-body object for U = 5.0 m/s: (**a**) square, (**b**) circle.

**Figure 16:**
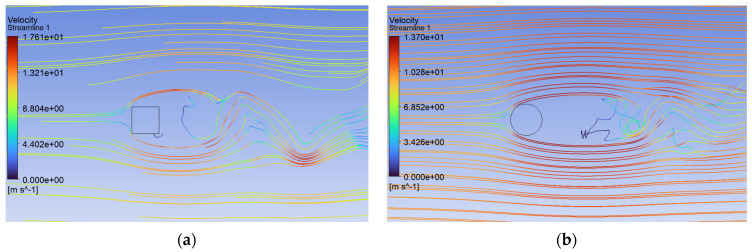
Streamlines around the examined fixed bluff-body object for U = 10.0 m/s: (**a**) square, (**b**) circle.

Analysis of the CFD simulation confirms that the values of forces acting on the tested object are influenced by the velocity distribution around it [51]. Figure 11, Figure 12 and Figure 13 show the velocity distributions in the longitudinal plane, while Figure 14, Figure 15 and Figure 16 show the streamlines. These are representative and correspond to three velocities (0.5, 5.0, 10.0 m/s) among all the airflow speeds for which the model was analyzed. For the square cross section, significantly higher local velocities were obtained compared to the circular cross section for the same condition at the inlet. For example, for v = 0.5 m/s, the speed in the case of the square cross section locally increased to a value of approximately 0.84 m/s, and in the case of the circular cross section to 0.68 m/s. For higher speeds, i.e., 5.0 m/s and 10 m/s, these differences were 10.27 m/s to 6.44 m/s and 15.82 m/s to 12.86 m/s, respectively.

**Figure 17:**
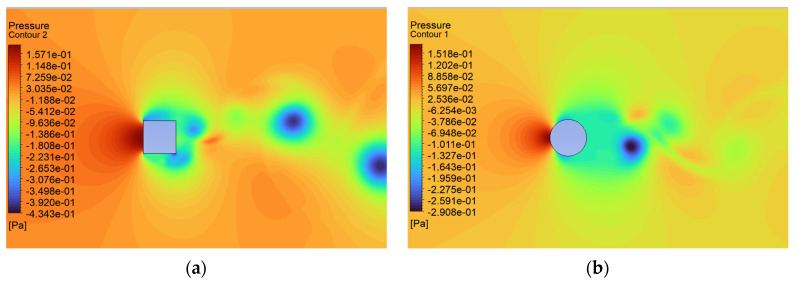
Pressure contour around the examined fixed bluff-body object for U = 0.5 m/s: (**a**) square, (**b**) circle.

**Figure 18:**
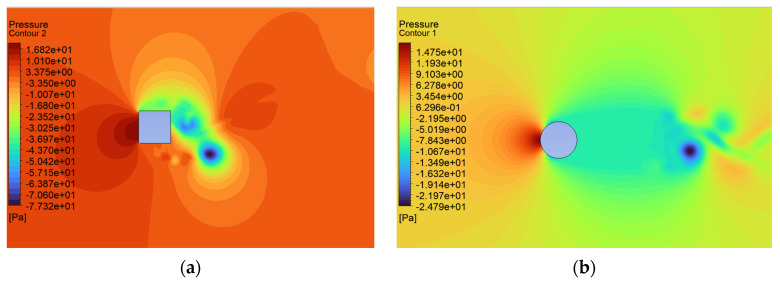
Pressure contour around the examined fixed bluff-body object for U = 5.0 m/s: (**a**) square, (**b**) circle.

**Figure 19:**
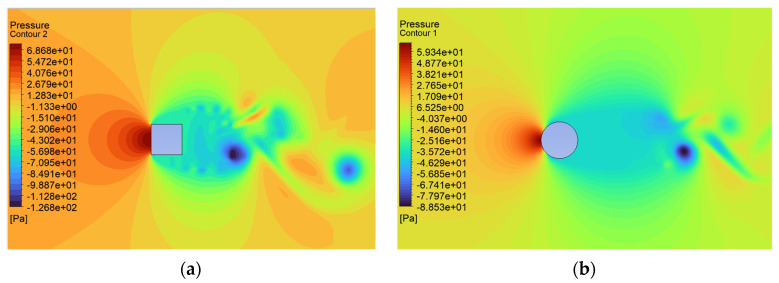
Pressure contour around the examined object fixed bluff-body object for U = 10.0 m/s: (**a**) square, (**b**) circle.

The consequence of the cases considered here leads to changes in the pressure distribution characteristic of these shapes (Figure 17, Figure 18 and Figure 19). The maximum local pressure occurred in both cases from the side of the fluid streamlining the object (from the front). As an example, for U = 0.5 m/s, the maximum frontal pressure value obtained was 0.1888 Pa and 0.1755 Pa for the square and circular sections, respectively. For U = 5 m/s, it was already 21.8600 Pa and 16.8650 Pa, and at a maximum speed of U = 10 m/s, it was 79.15 Pa and 67.2675 Pa. The pressure differences between the shapes considered ranged from 7% to 22%, taking into account the above values. Due to local changes in speed, the largest differences were observed in the form of negative pressure behind the object, which led to increased vorticity and forces acting in the direction perpendicular to the flow. In the case of the square cross section, the negative pressure for speeds of 0.5 m/s, 5.0 m/s, and 10.0 m/s was −0.43 Pa, −77.32 Pa, and −126.80 Pa, respectively. For the circular cross section, this was 0.29 Pa, −24.79 Pa, and −88.53 Pa, respectively.

Airflow around the square and circular cross sections differed significantly, which affected the distribution of speed and pressure, as well as the formation of vortices around and behind the object. The flow around the circular body was more symmetrical, and laminar flow (at low Reynolds numbers e.g., at v = 0.5 m/s) flowed around it more easily. At higher flow velocities, a zone of detached flow was created behind the object, but it was smaller than with the square cross section. Due to the gentle (rounded) flow, the air velocity around the circular cross section did not change dramatically. Square edges created greater flow disruption by creating flow separation zones at the edges. This caused significant changes in flow velocity, especially near corners. The flow behind the square cross section underwent more turbulent detachment and larger vortices being created compared to the circular cross section.

In the case of the circular cross section, the pressure was more evenly distributed around the body surface, which was the effect of gentle flow. The dynamic pressure decreased gradually as the flow broke away. On the front part (leading edge), there was a zone of higher pressure, while behind the body, there was a zone of lower pressure with a smaller gradient compared to the square cross section.

In the case of the square cross section, there was a clear high-pressure zone on the front wall that quickly dropped to low values on the side walls. As the air separated at sharp edges, a large low-pressure zone was created behind the object, leading to a greater pressure difference between the front and rear. The square corners resulted in local pressure increases and rapid drops behind them.

Behind the circular cross section, one can observe the phenomenon of stream detachment where Kármán vortices are formed. These vortices are less intense than in the case of the square cross section. Vortices form behind an object in a relatively predictable and symmetrical manner. In the case of the square cross section, the edges promoted faster detachment of the flow and more irregular formation of vortices. Due to the sharp edges, larger, more chaotic vortices were created, leading to stronger fluctuations in speed and pressure, greater turbulence, faster vortex formation, and more irregular forces acting on the object. This would make the square cross section more effective in energy harvesting applications when the goal is to maximize harvesting responses from the vibrations. This can also be effective when looking into extreme value estimates from harvesting or trying to establish a link with fragility curves [52]. There is a greater tendency for greater pressure fluctuations linked to vortex formation and forces that can cause vibrations. However, this comes at the cost of larger fluctuations in generated power, as well as higher fatigue on the vibrating object. The circular section generated more regular and predictable vortices, which may be beneficial in terms of fewer fluctuations and less stress on the host structure from which energy is being harvested, but came with less enhanced harvesting responses compared to the square section. Consequently, the needs of application and the allowable fluctuations guide the appropriate design choice for the harvester along with frequency and amplitude characteristics of the excitation, along with system efficiency.

**Figure 20:**
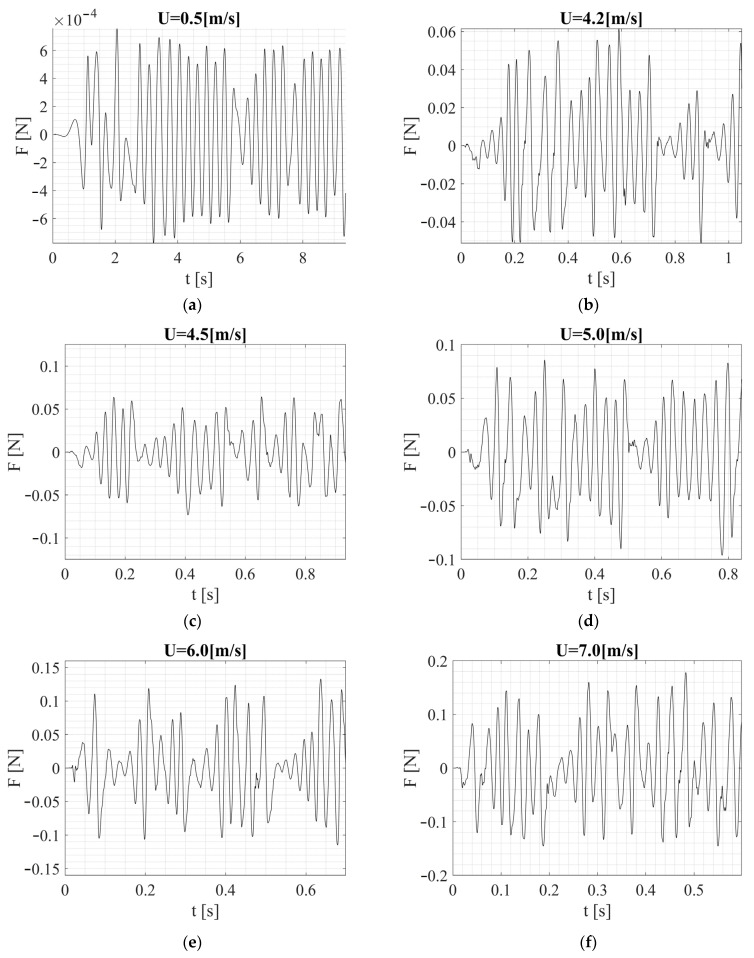

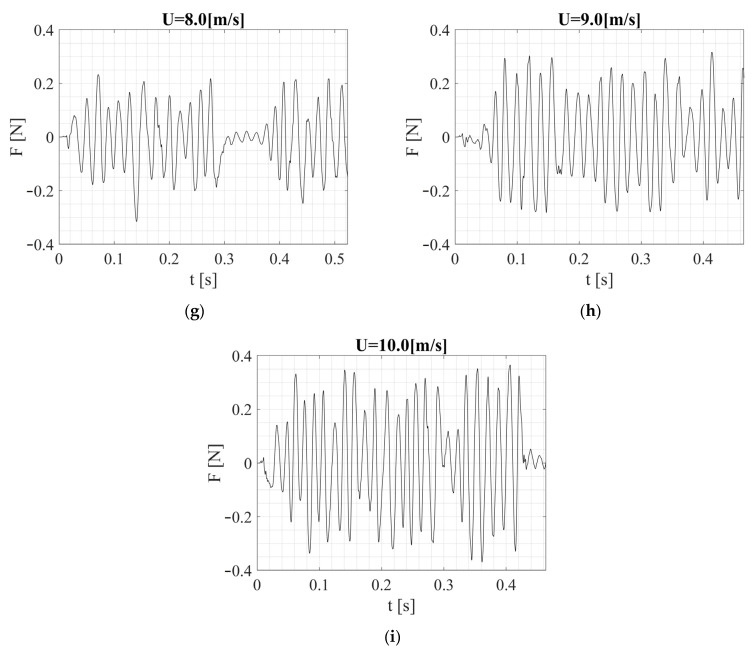
Forces acting on the fixed bluff-body (square cross section) by the specific value of the airflow velocity: (**a**) U = 0.5 m/s, (**b**) U = 4.2 m/s, (**c**) U = 4.5 m/s, (**d**) U = 5.0 m/s, (**e**) U = 6.0 m/s, (**f**) U = 7.0 m/s, (**g**) U = 8.0 m/s, (**h**) U = 9.0 m/s, (**i**) U = 10.0 m/s.

**Figure 21:**
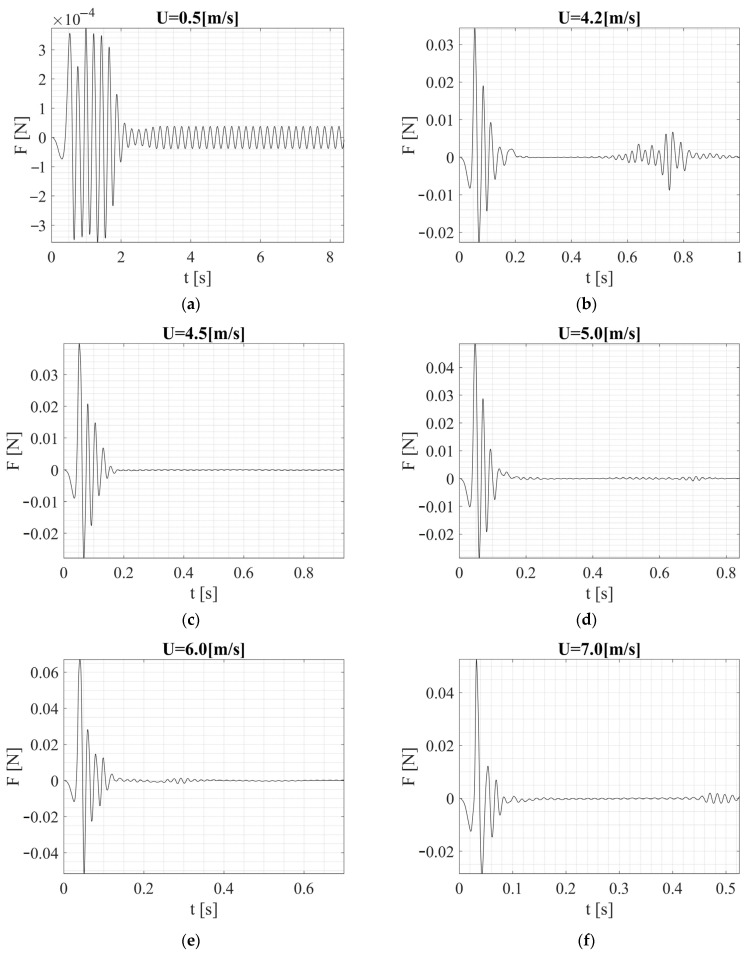

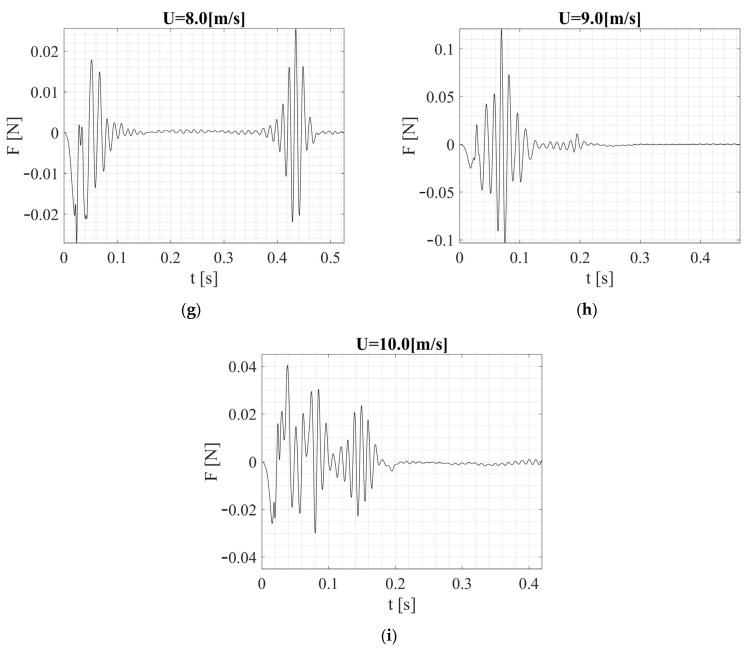
Forces acting on the fixed bluff-body (square circular-section) by the specific value of the airflow velocity: (**a**) U = 0.5 m/s, (**b**) U = 4.2 m/s, (**c**) U = 4.5 m/s, (**d**) U = 5.0 m/s, (**e**) U = 6.0 m/s, (**f**) U = 7.0 m/s, (**g**) U = 8.0 m/s, (**h**) U = 9.0 m/s, (**i**) U = 10.0 m/s.

Figure 20 and Figure 21 show the time histories of the lateral force acting on both shapes. The square section generated more irregular, but stronger force (velocity and pressure) fluctuations. This led to irregular vibrations with potentially greater amplitude. Stronger force fluctuations mean that the vibration system must be adapted to handle larger and even rapid changes in the driving force. This can lead to a wider vibration frequency band at the expense of higher stress and fatigue, along with energy losses in electromechanical circuits, but is more effective in systems that operate over a wide frequency range and are not optimized for a single dominant frequency. This can also be relevant for probing damages in testing structures, since the excitation bandwidth and responses are higher, making the harvester more efficient as a monitor by simply changing the excitation bandwidth through the design characteristics of the cross section. Additional damping or control for the square cross section structure may be required in some cases, but it also has the potential to reduce the harvested energy. The square cross section configuration will accentuate the harvested energy under variable flow rate conditions.

The circular cross section, due to its more symmetrical flow, generated more regular and predictable vibrations. Kármán eddies formed with greater regularity, leading to forcing with stable, oscillating characteristics. The vibration system can be tuned to one dominant frequency because the driving force signal is more regular. Therefore, the circular cross section is better suited to systems that are optimized to operate within a narrow range of resonant frequencies and applications requiring more smoothness in the harvesting signals. For model validation and system identification, this is useful when operating closer to a certain known frequency, and the results can be used for the model through their repeatability and for more precise measurement for a certain frequency of interest. For harvesting from inputs with higher variability and fluctuations, this cross section can be less effective than a square one.

In summary, the square section is a better choice when the goal is to generate irregular, intense, high-amplitude vibrations, which is beneficial in energy harvesting systems that convert energy from unstable flow fluctuations. The circular section is more suitable for vibrating systems that require stable, regular vibrations of lower amplitude and predictable frequency, which may be advantageous in precision or narrow-frequency systems. The choice of shape, therefore, depends on whether the priority is to maximize fluctuations and amplitude (square) or control over vibration stability and frequency (circular).

The analyses also indicated that combining the two shapes is not an optimal solution for the energy harvesting system. Consider the characteristics of the lateral forces acting on both shapes, which can overall be described by F(t) = ½ ρ∙U2A∙CL(t), which oscillates periodically around the axis of the cylinder or square beam. This force is the result of the pressure difference between the side where the vortex detaches first and the side where it detaches later. Although for a circular cross section, maximum CL values are assumed in the range of 0.5–1.00 for dynamic analyses such as VIV, in practice, the average values are lower, around 0.2. By substituting into the above dependence for, for example, the first case with U = 0.5 m/s, we can expect a lateral force of F = 3 × 10^−4^ N with CL equal to 1 and F = 6.13 × 10^−5^ N with CL equal to 0.2. These magnitudes should be taken into account to have an idea of the interpretation of the values obtained from the calculations. If we consider the maximum speed at which the research was conducted, i.e., 10 m/s, the force can be F = 1.23 × 10^−1^ N and F = 2.45 × 10^−2^ N, respectively. The results obtained through CFD had converging values, but it should be noted that at higher speeds, vortex-induced vibrations diminished, which is visible in Figure 21 and also linked to a more dispersed character of lateral forces. Regarding the square cross section, a different situation was observed. First, the CL coefficients reached slightly higher instantaneous values compared to the circular cross section, but most importantly, the amplitude of the force increased with speed within the tested range. This is related, among other things, to a different frequency of vortex generation, which in this case did not dissipate its energy as intensively as with the circular cross section. Considering the model used for the experiment, it should be emphasized that the shape along the beam was not purely circular or square and represented a smooth transition between them. The combination of these two shapes led to a more irregular flow, complicating the prediction of aerodynamic properties and causing mutual interference.

## 6. Conclusions

This paper considered the application of hybrid excitation and its effect on the performance of a piezoelectric energy harvesting system. Experiments conducted in this regard provide insights into the consequences of hybrid excitation. Broadband wind excitation with mechanical narrowband harmonic excitations was considered in the context of a wind tunnel experiment. A transition between a circular and a square cross section was considered for experiments, and the effects of circular and square cross sections were individually assessed numerically through CFD simulations.

Dual excitation in the form of harmonic mechanical inputs and airflow caused an increase in voltage by at least 27% for one of the masses compared to a single source of excitation. The maximum voltage could be doubled for the heaviest applied masses in the experiment. The additional positive effect of the application of hybrid excitation is the shift in the characteristic frequency of bluff bodies, which improves the flexibility of the system in terms of harvesting or probing a certain frequency range of the system via harvesting. Also, adding a combined excitation source allows for the system to operate in a wider frequency range and obtain more energy. The wind excitation source considered via CFD simulations focused on the effects of circular versus square cross sections and if a transition, as evidenced by the experiments, is an effective combination of the two. The flow mechanisms, vibrations, and the force distribution acting on the bluff-body as a function of time were also estimated, which made it possible to assess the impact of variable flow parameters on the efficiency of the energy harvesting system. It was observed that the enhanced energy harvesting from the square cross section came with the requirement to adjust the right amount of structural stresses or fatigue, possible electromechanical losses, and fluctuations, but also provided additional flexibility for the system, while a circular cross section led to more precision, repeatability, and less structural stress, but against lower harvested energy and also flexibility around the operational range of the harvester. It was also observed that a combination of the two was not often an efficient middle ground.

In the future, it is planned to extend the tests with the application of nonlinear springs [53] including mechanical barriers [54,55] and investigate the performance of these nonlinear systems in detail, along with their dynamic characteristics. What is worth mentioning is the possibility of using prototypes in various practical applications, such as in photovoltaics [56], maritime [57], polymers [58], or in isolators [59].

## Figures and Tables

**Table 1:** Data of shaker’s peak-to-peak displacement (*x*) in the domain of frequency of excitation (*f*).

No.	f (Hz)	x (mm)	No.	f (Hz)	x (mm)
1	0.5	0.72	19	2.825	3.56
2	1	1.34	20	2.85	3.62
3	1.5	1.62	21	2.875	3.73
4	2	1.83	22	2.9	3.81
5	2.2	1.92	23	3	3.93
6	2.25	2.13	24	3.25	4.12
7	2.3	2.21	25	3.3125	4.27
8	2.325	2.38	26	3.375	4.38
9	2.35	2.53	27	4.4375	4.42
10	2.4	2.61	28	3.5	4.51
11	2.5	2.72	29	3.625	4.64
12	2.55	2.85	30	3.75	4.71
13	2.6	2.96	31	3.825	4.83
14	2.625	3.22	32	3.9	4.97
15	2.65	3.14	33	4	5.01
16	2.675	3.21	34	4.5	5.24
17	2.7	3.34	35	5	5.49
18	2.8	3.42	36	-	-

**Table 2:** Physical data of bluff bodies with characteristic frequencies.

Infill Percentage (%)	Mass of Bluff-Body (g)	Characteristic Frequency (Hz)	Voltage (V)
20	15.7	3.75	4.35
40	21	3.3125	4.03
60	30.4	2.8	2.37
80	37.8	2.675	1.14
100	45.6	2.3	0.94

**Table 3:** Results of maximal output voltage by hybrid excitation.

Infill Percentage (%)	f (Hz)	U (m/s)	V (V)
20%	2	8	6.68
40%	3	7	5.12
60%	1	7.5	3.20
80%	2.6	7	2.56
100%	2.2	7.5	2.04

**Table 4:** Performance change by single and hybrid source of excitation.

Infill Percentage (%)	Shaker Excitation (V_rms_)	Hybrid Excitation (V_rms_)	Performance Increase
20	4.35	6.68	53.50%
40	4.03	5.12	27%
60	2.37	3.2	35%
80	1.14	2.56	125%
100	0.94	2.04	117%

**Table 5:** Changes in Reynolds number for different cross sections for all studied airflow velocities. In the column, the time step used in calculations is specified.

No.	v (m/s)	Re (-) (Circle)	Re (-) (Square)	Time Step (s)
1	0.5	809.02	717.21	0.004200
2	4.2	6795.7	6024.59	0.000500
3	4.5	7281.15	6454.92	0.000467
4	5.0	8090.16	7172.13	0.000420
5	6.0	9708.20	8606.56	0.000350
6	7.0	11,326.23	10,040.98	0.000300
7	8.0	12,944.26	11,475.41	0.000263
8	9.0	14,562.30	12,909.84	0.000233
9	10.0	16,180.33	14,344.26	0.000210

## Data Availability

Data are available on request due to privacy restrictions.
